# Argentine Black and White Tegu (*Salvator merianae*) can survive the winter under semi-natural conditions well beyond their current invasive range

**DOI:** 10.1371/journal.pone.0245877

**Published:** 2021-03-10

**Authors:** Scott M. Goetz, David A. Steen, Melissa A. Miller, Craig Guyer, Jack Kottwitz, John F. Roberts, Emmett Blankenship, Phillip R. Pearson, Daniel A. Warner, Robert N. Reed

**Affiliations:** 1 U.S. Geological Survey, Fort Collins Science Center, Fort Collins, Colorado, United States of America; 2 Fish and Wildlife Research Institute, Florida Fish and Wildlife Conservation Commission, Gainesville, Florida, United States of America; 3 Department of Wildlife Ecology and Conservation, Fort Lauderdale Research and Education Center, University of Florida, Davie, Florida, United States of America; 4 Department of Biological Sciences, College of Sciences and Mathematics, Auburn University, Auburn, Alabama, United States of America; 5 Department of Anatomy, Physiology, and Pharmacology, College of Veterinary Medicine, Auburn University, Auburn, Alabama, United States of America; 6 Department of Comparative, Diagnostic, and Population Medicine, College of Veterinary Medicine, University of Florida, Gainesville, Florida, United States of America; 7 All Pets Emergency and Referral Center, Alpharetta, Georgia, United States of America; 8 Centre for Conservation, Ecology, and Genetics, Institute for Applied Ecology, University of Canberra, Bruce, Australian Capital City, Australia; University of Regina, CANADA

## Abstract

The Argentine Black and White Tegu (*Salvator merianae*, formerly *Tupinambis merianae*) is a large lizard from South America. Now established and invasive in southern Florida, and it poses threats to populations of many native species. Models suggest much of the southern United States may contain suitable temperature regimes for this species, yet there is considerable uncertainty regarding either the potential for range expansion northward out of tropical and subtropical zones or the potential for the species establishing elsewhere following additional independent introductions. We evaluated survival, body temperature, duration and timing of winter dormancy, and health of wild-caught tegus from southern Florida held in semi-natural enclosures for over a year in Auburn, Alabama (> 900 km northwest of capture location). Nine of twelve lizards emerged from winter dormancy and seven survived the greater-than-one-year duration of the study. Average length of dormancy (176 d) was greater than that reported in the native range or for invasive populations in southern Florida and females remained dormant longer than males. Tegus grew rapidly throughout the study and the presence of sperm in the testes of males and previtellogenic or early vitellogenic follicles in female ovaries at the end of our study suggest the animals would have been capable of reproduction the following spring. The survival and overall health of the majority of adult tegus in our study suggests weather and climate patterns are unlikely to prevent survival following introduction in many areas of the United States far from their current invasive range.

## Introduction

Biological invasions increase global biotic homogeneity [1,2], threaten biodiversity [3,4], and may inflict severe ecological and economic impacts [5,6]. Nonindigenous species, however, must first pass through a series of invasion stages before impacting recipient ecosystems [7]. Moreover, ecological effects and control cost often scale with the total size of occupied area. Thus, proactive management approaches that inhibit the initial stages of the invasion process are essential. Introductions can be avoided by intercepting organisms during the transport stage, but this is problematic for species that are popular in the pet trade, legally imported and owned, and easily bred in captivity; three factors common to many species of exotic reptiles and amphibians in the United States. Without the ability to control the transport of organisms, establishment (i.e. a reproductive population) can still be prevented through early detection of incipient populations and rapid response as the likelihood of successful eradication is negatively correlated with time since introduction [8]. However, detection of newly introduced or emerging populations is difficult because many nonindigenous species remain unnoticed until after they are firmly established, and after small-scale eradication efforts are no longer viable. Clearly, it is difficult and costly to detect and manage invasions after they have occurred. Proactive efforts are advisable, but there is often limited information regarding which areas contain conditions conducive to establishment.

History of extralimital establishment is one of the few reliable cross-taxa predictors of invasion success [9–11] and can help inform whether a nonindigenous species may be likely to establish secondary populations if introduced elsewhere. Moreover, models of bioinvasions consisting of multiple, independent populations may be best managed by focusing control efforts on satellite populations rather than on the initial, often larger, population [12,13]. Viewed through the lens of proactive management, these models can be interpreted to suggest that the best approach is to prioritize containment of the founding population to prevent the formation of secondary populations. Species distribution models provide a valuable tool to delineate the potential geoclimatic limits of an invasion and identify areas where establishment is most likely. However, experimental testing is necessary to fully understand how an organism will respond to climatic regimes identified by models as suitable. For example, modeling exercises related to an invasive population of a large reptile in southern Florida, Burmese Pythons (*Python bivittatus*), resulted in widely disparate results [14–16]; corroborative data obtained by experimentally evaluating survival of pythons under semi-natural conditions outside of their introduced range [17] helped clarify conflicting results obtained through modeling.

The Argentine Black and White Tegu (*Salvator merianae*, formerly *Tupinambis merianae*) is native to South America, but is now established in Hillsborough, Polk, and Miami-Dade counties in Florida, USA [18–20], and an island off the Brazilian coast [21]. Recent findings also suggest an established population in southern Georgia [22]. Tegus in the adjacent Florida counties of Hillsborough and Polk are separated from those in Miami-Dade County by approximately 300 km. There is little evidence of gene flow between the two Florida populations [23], but both appear to have resulted from escaped or released individuals from the pet trade in the early 2000s [18,19,23]. Thus, tegus have twice successfully established populations in Florida where they pose a threat to native and imperiled species [24,25]. Active control efforts are underway and over 3,300 tegus have been removed from Miami-Dade County in the past ten years [26], but tremendous effort is likely needed to achieve significant reductions in the population [27]. Population resilience in the face of removal efforts is unsurprising given tegu populations in their native range have historically withstood substantial harvests for the leather trade [28].

Species distribution models based on occurrence in the native range indicate that suitable conditions for the Argentine Black and White Tegu may occur over a large portion of the southern United States [29]. A climate match alone cannot predict establishment of nonindigenous species, and tegus possess many additional traits that are positively associated with invasion success, such as early maturity, annual reproduction, large clutch size, and a relatively long life span [28,30,31]. Tegus attain the largest body sizes (to ~5 kg) of any members of the family Teiidae [32], and a wide dietary breadth (fruits, invertebrates, small vertebrates, eggs, and carrion [24,33–36]) may also contribute to their success outside their native habitat. Tegu behavioral ecology may further facilitate survival and expansion in the southern United States. First, tegus occupy a variety of forested and open habitats, including disturbed areas [37–39], such that habitat structure is unlikely to impede invasive range expansion. Second, tegus are seasonally active and escape cold or dry climatic conditions by hibernating in underground refugia [32,40,41]; a response that likely increases survival in regions that routinely experience moderately cold winter conditions. Finally, tegus are capable of elevating their body temperature through a form of endothermy, which may promote survival at relatively low temperatures [42]. Collectively, the biology of tegus suggests there is little reason to suspect invasions in the continental United States will be limited to southern Florida.

Here, we evaluated the ability of tegus to survive for a year in a temperate climate within the bounds of suitable temperature regimes identified by climate-matching models [29]. Our primary objective was to assess whether tegus could survive a winter season in which surface air temperatures typically drop below freezing in a semi-natural setting. Specifically, we assessed the effect of sex on tegu survival, body temperature, length of winter dormancy, date of emergence from dormancy as well as body temperature in relation to surface air temperature. We monitored the general health of tegus to investigate potential detrimental effects of climate and assessed progression towards reproductive maturity. We provide a qualitative comparison of average daily high and low winter temperatures of our study site with invaded regions of Florida and the southern limit of the tegu native range to add context to our findings.

## Methods

### Study subjects, experimental enclosures, and experimental design

Between 18 May and 27 June 2017, Argentine Black and White Tegus were live trapped (Havahart^®^, Lancaster, PA, USA) in Miami-Dade County, Florida (see S1 Table for exact capture locations). The 12 largest individuals that provided an adequate sample of each sex (8 males, 4 females) were selected for this study to allow investigation of sex effects and reproduction following hibernation. However, only one male was sexually mature at time of capture and all other individuals were slightly below minimum size estimates for sexual maturity of the Argentine Black and White Tegu and the closely related Red Tegu (*Salvator rufescens*) [26,43]. At time of capture, females had an average mass of 647.0 ± 33.0 g (mean ± 1 SE; range = 590–734 g) and an average snout-vent length (SVL) of 279.3 mm (mean; range = 270–295 mm; exact length data not available for one female but SVL was within stated range) and males had an average mass of 1,047.8 ± 244.3 g (range = 610–2720 g) and an average SVL of 299.1 ± 13.8 mm (range = 270–392 mm). Within two weeks of capture, between 25 May and 06 July 2017, tegus were transported to Auburn University, Auburn, Lee County, Alabama where they were initially housed in outdoor enclosures measuring 3.65 m long by 1.22 m wide by 2.13 m in height with concrete floors and fully enclosed with hardware cloth. Enclosures included a large water bath (63.5 cm x 43.2 cm wide x 22.9 cm deep) allowing constant access to water, a bale of pine straw for natural cover, and a piece of plywood propped on its side to serve as a refuge. We implanted an internal radio transmitter (11 g; Model R1530, Advanced Telemetry Systems, Isanti, MN, USA) in each individual on 03 August 2017 as a biosecurity measure in the event of an escape. We also implanted internal thermal micro dataloggers (© iButton Thermochrons) set to record body temperatures (T_b_) every 65 min from 03 August 2017 to 10 June 2018 (detailed surgical procedures provided in S1 Appendix).

**Fig 1:**
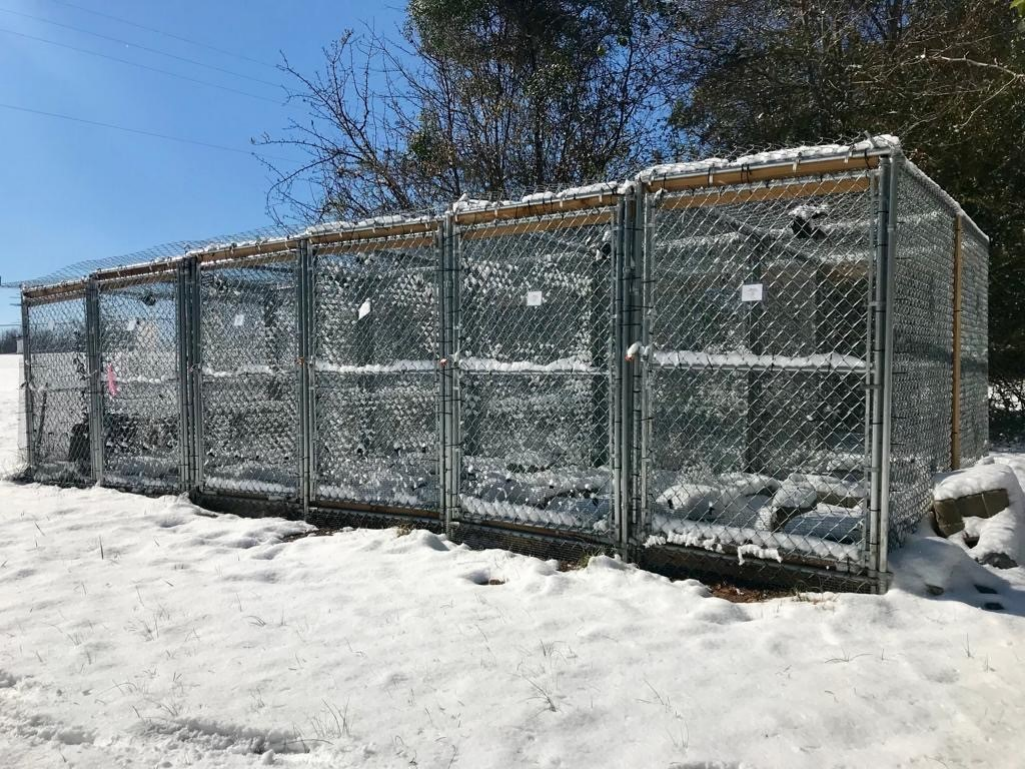
Outdoor soil-floor tegu enclosures in Auburn, AL. Tegus were housed in these enclosures during the late activity, winter dormancy, and early activity periods (22 August 2017–20 April 2018). Each enclosure contained an artificial refugia box buried 1 m underground and connected to the surface with corrugated drainpipe, pine straw spread on the ground, and concrete blocks lining the perimeter. Photograph taken on 17 January 2018 by MAM following an abnormal accumulating snow event in Auburn, AL.

On 22 August 2017, tegus were moved to individual outdoor enclosures with a soil floor where they remained throughout the ensuing winter and early spring (Fig 1). Enclosures measured 2 m length by 1.3 m width by 2 m height and were completely enclosed in chain link fencing buried 0.5 m underground. Chicken wire was attached to the interior surfaces of the fencing to prevent animals from exiting through the holes in the chain link, and concrete blocks were staged along all interior edges of enclosures to discourage digging and provide basking sites. In each enclosure, we installed an artificial refugium consisting of a 0.6 m x 0.2 m wooden box buried 1 m below the surface. The refugium was connected to the surface with a 2 m length of 10.2 cm diameter corrugated drainpipe bent in a U-shape to reduce air drafts and almost entirely buried to prevent heat conduction generated by solar radiation on the black pipe. We placed three micro dataloggers (iButton Thermochrons) in each enclosure set to record temperatures every 180 min from 22 August 2017 to 20 April 2018. Within each enclosure, dataloggers were placed in the following locations: shaded areas, unshaded areas, and on the floor of underground refugium. Specifically, dataloggers were affixed with silicone to the underside (shaded) or top (unshaded) surface of concrete blocks or the floor of refugia boxes. A bale of pine straw was scattered around each enclosure to provide additional natural cover that the tegus could also use to insulate burrows. Each enclosure contained a water bath relocated from concrete-floor enclosures allowing constant access to water in a container large enough to permit tegus to fully immerse their bodies. From the ceiling of each enclosure we hung an automated, motion-sensitive trail camera (Reconyx, Holmen, WI, USA) to monitor tegu activities and behavior. Twice a week each tegu was offered a diet consisting of commercially purchased frozen/thawed mice, quail, or hardboiled chicken eggs as well as a variety of locally available fruits and vegetables. When a tegu remained underground for more than seven days, we considered that lizard to be in hibernation and stopped offering food until the individual was observed outside the burrow in February 2018 or later.

On 20 April 2018, we began returning lizards to the larger concrete-floor enclosures where they were originally housed upon arrival to accommodate the housing of pairs of tegus in the same enclosure and for ease of monitoring behavior. After all lizards were transferred, we performed a general health assessment and weighed each animal. Tegus were housed as male-female pairs to evaluate whether they exhibited behaviors consistent with reproduction and females were rotated to expose them to multiple males.

Surviving lizards were euthanized at the end of the study (see S1 Appendix). Full necropsies were performed by coauthors JK and JFR on all lizards that died during the study or survived until the end of the study. Necropsies included collection of morphological data (mass, SVL, fat pad mass, liver mass), assessment of reproductive status and potential (males: formalin fixed testicle mass, length, height, and width, seminiferous tubule width, and estimated number of spermatocytes; females: ovary mass), and overall health (e.g., evidence of gross abnormalities, disease, or parasites) at time of death.

This study was conducted in strict accordance with the recommendations and protocol approval of the Auburn University Institutional Animal Care and Use Committee (IACUC Protocol 2017–3086). All surgery was performed under propofol and isoflurane gas anesthesia (see S1 Appendix), and all efforts were made to minimize suffering. Collection of Argentine Black and White Tegus was approved under a Florida Fish and Wildlife Conservation Commission permit (EXOT-17-05a).

### Analyses

We defined four biologically relevant time periods as a framework to evaluate aspects of tegu survival and behavior. These periods included late active (11 weeks prior to the last individual to enter dormancy), dormancy (hibernation; interval between last individual to enter dormancy and first individual to emerge; 17 October– 22 February), early active (8 weeks post first individual to emerge), and active (between early and late active). For each time period, we calculated average, minimum, and maximum tegu T_b_ including only temperatures of individuals not hibernating during the early and late activity periods and only temperatures of individuals that survived in the dormancy period. We defined the start of dormancy for each individual as the first day a lizard was not observed on the surface and remained underground for over a week during which T_b_ exhibited a pattern inconsistent with basking behavior. We defined emergence from dormancy when an individual was seen on the surface more than three times in a week and/or T_b_ fluctuated in a manner consistent with basking behavior. For each time period, we calculated average, minimum, and maximum tegu T_b_ including only temperatures of individuals not hibernating during the early and late activity periods.

We used general linear models or Student’s *t* tests to investigate relationships between surface air temperature and T_b_ during the dormancy period and tegu survival or length of dormancy as a factor of mass. We used one-tailed Student’s *t* tests to evaluate the effect of sex on length of dormancy and date of emergence with the a priori prediction that males would emerge earlier and have a shorter hibernation period. We used repeated measures linear mixed effects models to investigate differences in tegu T_b_ as a factor of sex during both dormant and active periods and compared tegu T_b_ to shaded air surface temperatures during dormancy, early active, and late active periods. Models included the random effect of individual datalogger (internally implanted in lizards or staged in the environment) and a correlation argument to account for autocorrelation. Differences among factors were considered statistically significant at the level of P < 0.05 and statistical analyses were performed in R v3.6.1 [44].

Multiyear thermal regimes of our study site and locations within the native or invasive range were qualitatively described in two ways to provide context of the climatic suitability of our study site. First, we compared the ten-year average daily high and low winter temperatures for our study site, two Florida counties where tegus are established, and a location near the southern limit of the native tegu distribution in Argentina. To do so, we accessed 10 years (2007–2016) of climate data from the National Oceanic and Atmospheric Administration [45] for Lee County, Alabama, USA (Station USC00010425), Hillsborough County, Florida, USA (Station USC00087205), Miami-Dade County, Florida, USA (Station USC00084095), and Santa Rosa, Argentina (Station AR000087623) near the southern limit of the tegu native range [46]. Winter was defined as 21 December– 20 March for the three North American sites and 21 June– 20 September for the South American location. Second, using weather station data for Lee County, AL, we also calculated 95% confidence intervals for the daily ten-year average high and low temperatures and overlaid the temperatures recorded during the year of our study to assess variation from expected temperatures.

## Results

Nine of twelve tegus (75%) survived the winter and emerged from dormancy; seven tegus (58%) survived for a full year in outdoor enclosures in Auburn, Alabama, including three of four females and four of eight males (Table 1). Of the five tegus that died during the study, two individuals emerged from the winter dormancy period in the spring but later died (two and six weeks post emergence, including the largest and smallest male in the study, respectively). Three individuals were found dead in their artificial refugia after they failed to emerge in the spring.

**Table 1:** Tegu (*Salvator merianae*) morphological data, behavior, and fate.

Sex	Date Captured (2017)	Initial SVL (cm)	Initial Mass (g)	Ingress Date (2017)	Egress Date (2018)	Hibernation Survival	Length of Hibernation (days)	Cause of Death
F	29-May	27	590	22-Aug	23-Apr	Y	244	Euthanized at study end, 23 August 2018.
F	21-Jun	29.5	662	18-Sep	—	N	—	Undetermined, found dead in underground refugia. Carcass too decomposed for necropsy.
F	16-Jun	—	734	30-Sep	10-Mar	Y	161	Euthanized at study end, 23 August 2018.
F	27-Jun	—	—	5-Sep	20-Mar	Y	196	Euthanized at study end, 23 August 2018.
M	30-May	27	610	23-Aug	7-Mar	N	196	Found dead on 30-March-2018. Necropsy revealed respiratory infection.
M	10-Jun	29	694	15-Oct	15-Mar	Y	151	Euthanized at study end, 23 August 2018.
M	31-May	28.2	740	16-Oct	18-Mar	Y	153	Euthanized at study end, 23 August 2018.
M	21-May	27.7	764	28-Sep	22-Feb	Y	147	Euthanized at study end, 23 August 2018.
M	23-May	29	840	12-Oct	19-Mar	Y	158	Euthanized at study end, 23 August 2018.
M	9-Jun	31.5	946	22-Aug	—	N	—	Undetermined, found dead in underground refugia. Carcass too decomposed for necropsy.
M	22-May	29.3	1068	12-Oct	—	N	—	Undetermined, found dead in underground refugia. Carcass too decomposed for necropsy.
M	18-May	39.2	2720	2-Sep	28-Feb	N	179	Found dead on 18-April-2018. Necropsy revealed respiratory infection.

Description of individual lizard size, sex, date captured, behavior related to dormancy, and cause of death for the twelve Argentine Black and White Tegus (*Salvator merianae*) included in the study. SVL, snout-vent length.

**Fig 2:**
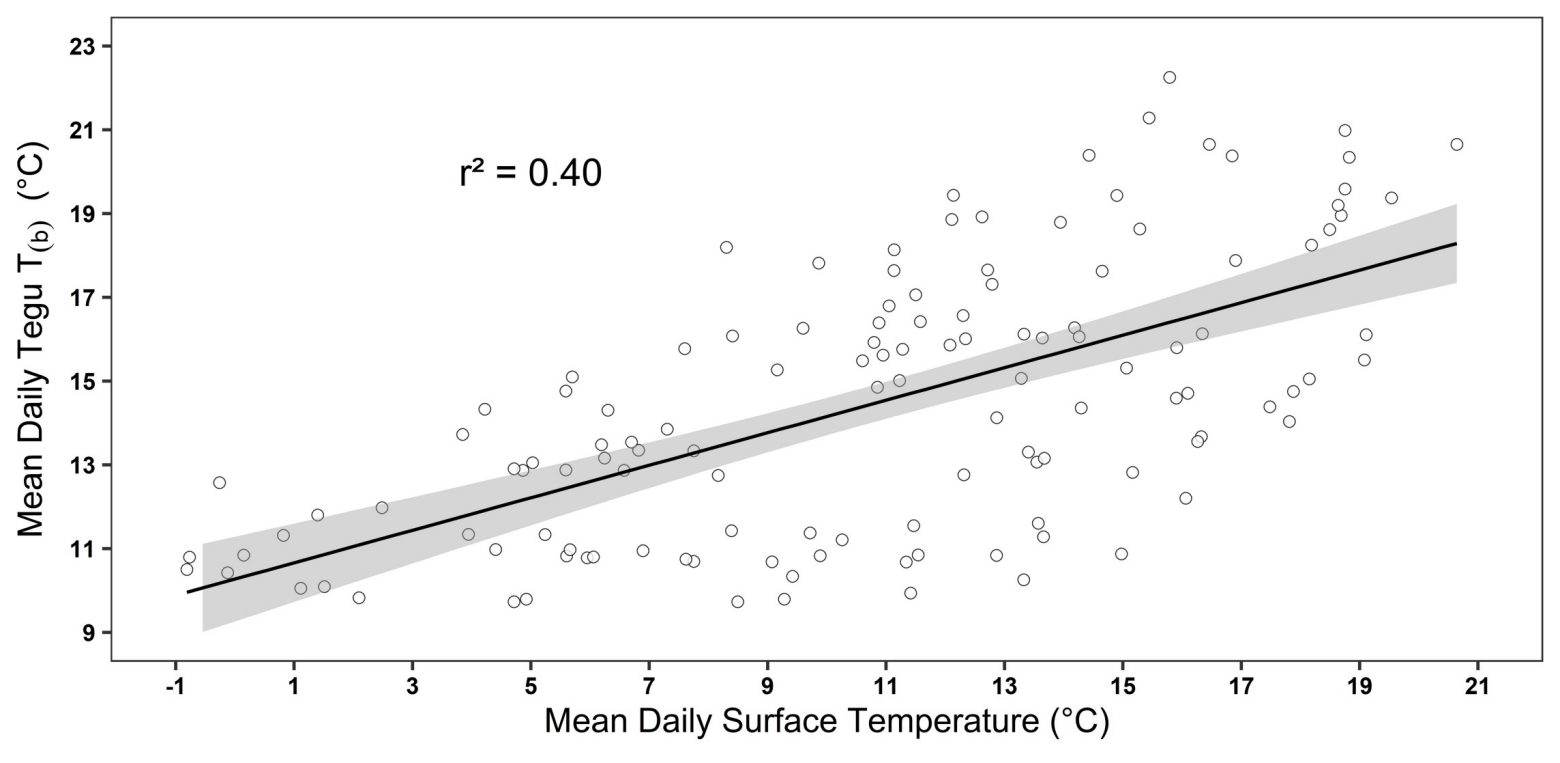
The effect of mean daily surface air temperature on tegu (*Salvator merianae*) mean daily body temperature during hibernation. Tegu mean daily body temperature (black line) surrounded by 95% confidence intervals (gray band) during the dormancy period (17 October 2017–22 February 2018). Each daily temperature (hollow circle) is the average of 11 iButton dataloggers placed in a shaded area of each tegu enclosure and internally implanted in 11 tegus (datalogger malfunctioned in tegu Sm-12).

**Fig 3:**
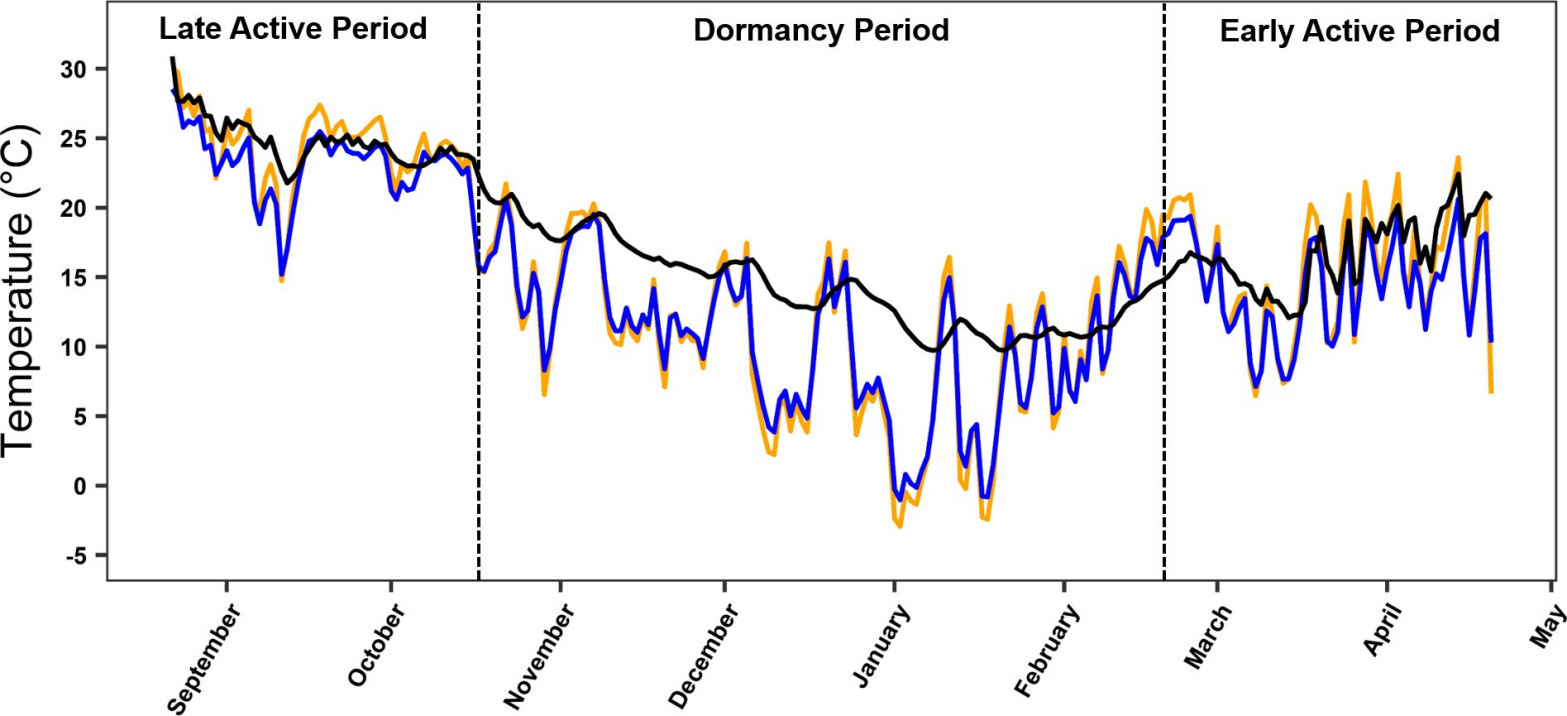
Mean daily tegu (*Salvator merianae*) body temperature in comparison to mean daily air temperatures. Mean daily body temperature of tegus (T_b_, solid black line; N = 11 internally-implanted micro dataloggers) and mean daily air temperature of micro dataloggers in shaded areas (blue line, N = 11) and unshaded areas (orange line; N = 11). The two vertical dotted lines define activity periods.

**Fig 4:**
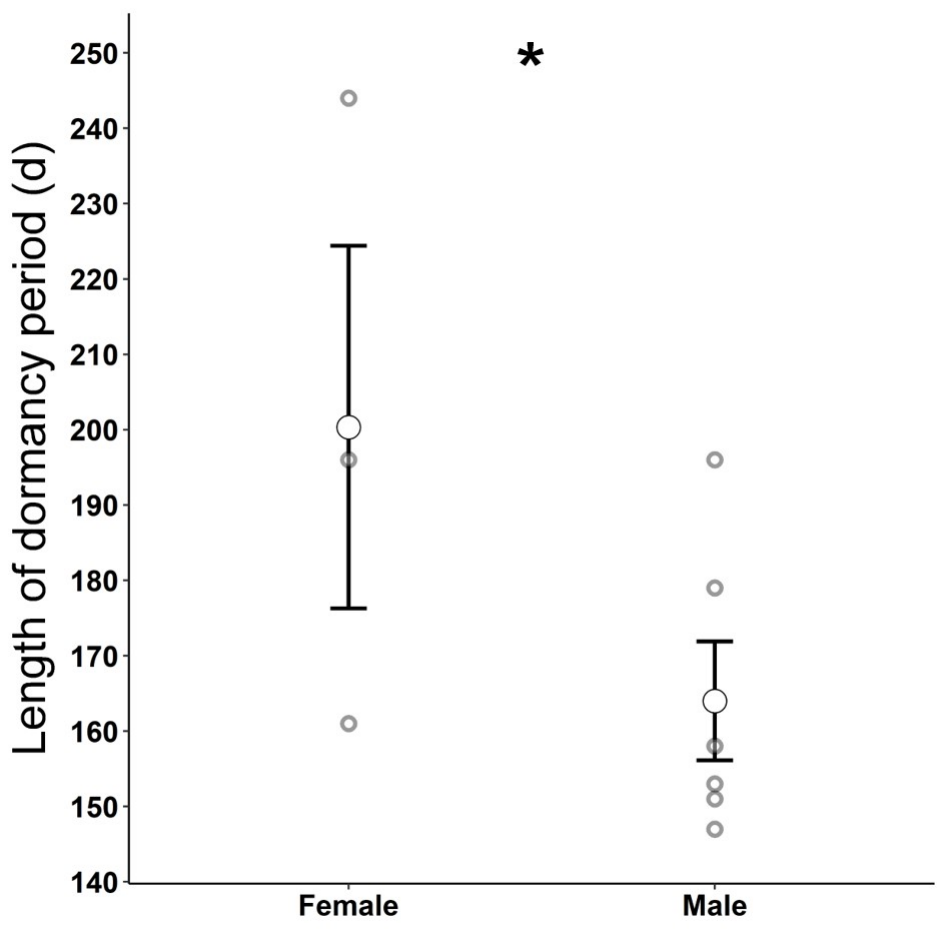
Sex specific mean length of tegu dormancy. Mean length of dormancy (open circles) of three female and six male Argentine Black and White Tegus (*Salvator merianae*) with error bars representing the standard error of the mean. Gray circles show length of dormancy for individuals and asterisk indicates statistically significant difference among sexes.

Average T_b_ during the dormancy period was 14.4°C ± 0.1, average maximum T_b_ was 14.7°C ± 0.1 (range = 5.1–26.6°C), and average minimum T_b_ was 14.2°C ± 0.1 (range = 4.6–23.6°C). Dataloggers placed in underground refugia failed to capture temperature data because of a programming error. However, during the dormancy period, we found that for each 1°C increase in mean daily shaded air temperature, we observed a 0.38°C (± 0.08, 95% CI) increase in mean daily tegu T_b_ (*F*_(1,127)_ = 83.41, r^2^ = 0.40, *p* < 0.001, Fig 2) such that fluctuations in aboveground air temperatures were reflected by smaller shifts in tegu T_b_ (Fig 3). Lizard body temperature was on average 3.07°C (SE = 0.532) warmer than shaded surface air temperatures and this effect was significant in the mixed effects linear model (*t*_(18, 2540)_ = 6.15, *p* < 0.001). Females exhibited a 0.15°C (SE = 1.368) higher average body temperature than males during the dormancy period but this effect was not significant in the mixed effects linear model (*t*_(7, 1143)_ = -0.116, *p* = 0.911). Females remained dormant for longer than males and the effect approached significance in the one-tailed Student’s *t* test (*t*_(7)_ = 1.860, *p* = 0.052, Fig 4). Average dormancy period for all lizards that emerged in the spring was 176 d ± 10.5 (range = 147–244 d) with an average female dormancy of 200 d ±24.1 (range = 161–244 d) and an average male dormancy of 164 d ±7.8 (range = 147–196 d). Males emerged from dormancy an average of 19.3 d earlier than females and the effect approached significance in the one-tailed Student’s *t* test (*t*_(7)_ = 1.801, *p* = 0.058). Student’s *t* tests revealed no relationship between mass and duration of dormancy (*t*_(8)_ = -0.058, *p* = 0.955) or survival and mass at time of capture (*t*_(11)_ = 0.161, *p* = 0.876).

Tegu behavior during non-dormancy periods was characterized by active exploration of enclosures and routine basking in sunlight during favorable weather conditions. We observed tegus responding to weather conditions unsuitable for basking, such as bouts of colder air temperatures or rain, by retreating and remaining underground. Lizard body temperature was on average 2.85°C (SE = 0.830) warmer than shaded surface air temperatures and this effect was significant in the mixed effects linear model (*t*_(18, 1823)_ = 3.442, *p* = 0.003). Males exhibited a 0.24°C (SE = 1.296) higher average body temperature than females during the non-dormancy periods but this difference was not significant in the mixed effects linear model (*t*_(7, 569)_ = 0.189, *p* = 0.855). During the late activity season (just prior to dormancy), average tegu T_b_ was 27.1°C ± 0.1 (mean ± SE), average maximum T_b_ was 32.5°C ± 0.2 (range = 19.6–40.5°C), and average minimum T_b_ was 25.0°C ± 0.1 (range = 19.1–29.1°C). Tegus began to hibernate between 22 August and 16 October, a range of 55 d.

The nine surviving lizards entering the early activity period following emergence from underground refugia did so between 22 February and 23 April, a range of 60 d. Average T_b_ for early activity period was 17.7°C ± 0.2 (mean ± SE), average daily maximum T_b_ was 27.1°C ± 0.5 (range = 10.6–39.1°C), and average daily minimum T_b_ was 14.3°C ± 0.1 (range = 5.1–19.1°C). Veterinary physical exams of seven surviving lizards at the end of the early activity period revealed no indication of disease or injury and all animals demonstrated alert behavior and normal physiologic parameters. By the end of the early active period, surviving lizards had gained an average of 336.7 g (± 63.0; range = 138–573 g) in mass since their initial capture representing an average increase of 48%.

At the beginning of the active period, we observed no aggression, courtship, or mating behavior between paired individuals and no females produced eggs. During the active period, average T_b_ was 27.2°C ± 0.2 (mean ± SE), average maximum T_b_ was 33.9°C ± 0.3 (range = 16.6–39.1°C), and average minimum T_b_ was 23.5°C ± 0.2 (range = 15.1–31.1°C). Necropsies following euthanasia, 23 August 2018, revealed the seven surviving lizards gained an average of 1,313.0 g (± 100.6; range = 991–1,681) in mass since their initial capture representing an average 186% increase. Gross and microscopic necropsy of these lizards did not reveal significant debilitating disease, and all had relatively large fat pads (Table 2). One female was found to have a commensal infection by a nonindigenous pentastome endoparasite, *Raillietiella orientalis*. Male testicle mass and the presence of mature spermatozoa and female ovary mass, with previtellogenic follicles (Table 2, S1 Fig), indicate all lizards were sexually mature by the end of the study following rapid growth during the just completed summer active period. Necropsies of the two males that died during the early activity period revealed heterophilic and granulomatous pneumonia with heavy lung growth of the bacteria *Serratia marcescens*, or moderate growth of the bacteria *Aeromonas hydrophilia* and heavy growth of the bacteria *Aeromonas veronii*, respectively. Decomposition was too advanced for the three individuals (2 males, 1 female) found dead in their artificial refugia boxes to allow a conclusive necropsy. During excavation of artificial refugia boxes in the spring, we observed two contained standing water and a thick layer of silt, corresponding to individuals that died during the dormancy period. Two of the 12 lizards refused food for the entirety of the study and both died during dormancy or shortly thereafter.

**Table 2:** Morphology data of surviving tegus (*Salvator merianae*). Characterization of growth and morphology data for the seven of twelve Argentine Black and White Tegus (*Salvator merianae*) that were captured in May or June 2017, survived a full year in semi-natural enclosures in Auburn, AL and were euthanized on 23 August 2018.

Sex	% Δ Mass Egress^1^	% Δ Mass Euthanasia^2^	% Δ SVL Euthanasia^3^	Fat Pad (g)	Liver Mass (g)	Testicle Mass^4^ (g)	TL (mm)	TH (mm)	TW (mm)	STD (μm)	Spermatocyte Number	Ovary Mass^4^ (g)
F	42	191	24	184	38	—	—	—	—	—	—	1.13
F	-	-	-	192	-	—	—	—	—	—	—	1.13
F	56	135	-	194	37	—	—	—	—	—	—	0.87
M	75	242	29	173	52	0.56	15	10	7	136	Moderate	—
M	35	186	30	175	47	0.46	12.5	7	8	85.1	Few	—
M	77	218	35	183	45	0.62	14	10	10	67.5	Few	—
M	16	151	28	211	42	0.27	13	6	7	72.3	Few	—

^1^Percent change in mass between initial capture and 17 May 2018

^2^Percent change in mass between initial capture and euthanasia on 23-Aug-2018

^3^Percent change in snout-vent length between initial capture and euthanasia on 23 August 2018

^4^Formalin fixed mass; TL, Testicle length; TH, Testicle height; TW, Testicle width; STD, Seminiferous tubule width.

**Fig 5:**
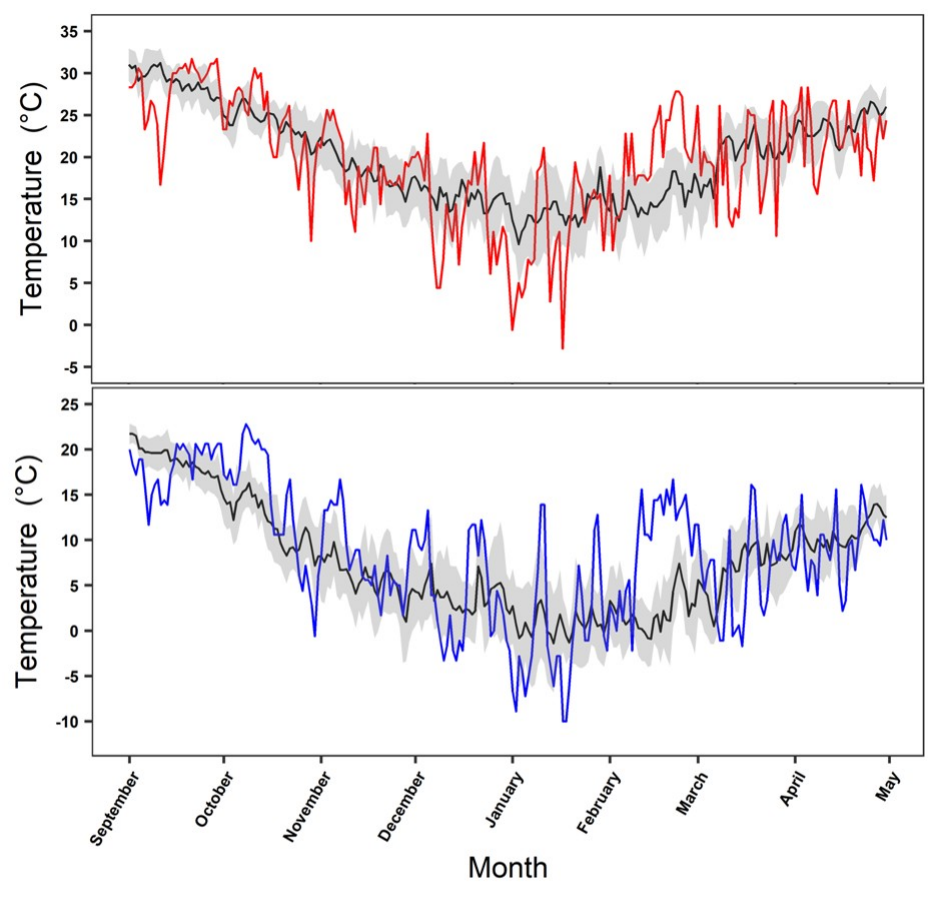
Comparison of air temperatures during the year of our study and the ten-year average. Mean ten-year (2007–2016) daily high (top panel) and low (bottom panel) temperatures from 01 September through 30 April for Lee County, AL (black line, top and bottom panel) and daily high (red line, top panel) and low (blue line, bottom panel) temperatures for this location during the year of our study (2017–2018). Gray band represents the 95% confidence for the ten-year average in both panels. Climate data retrieved from the National Oceanic and Atmospheric Administration (NOAA; https://www.ncdc.noaa.gov/cdo-web/datatools/selectlocation) in January 2020 from a weather stations in Lee County, Alabama (Station USC00010425).

**Fig 6:**
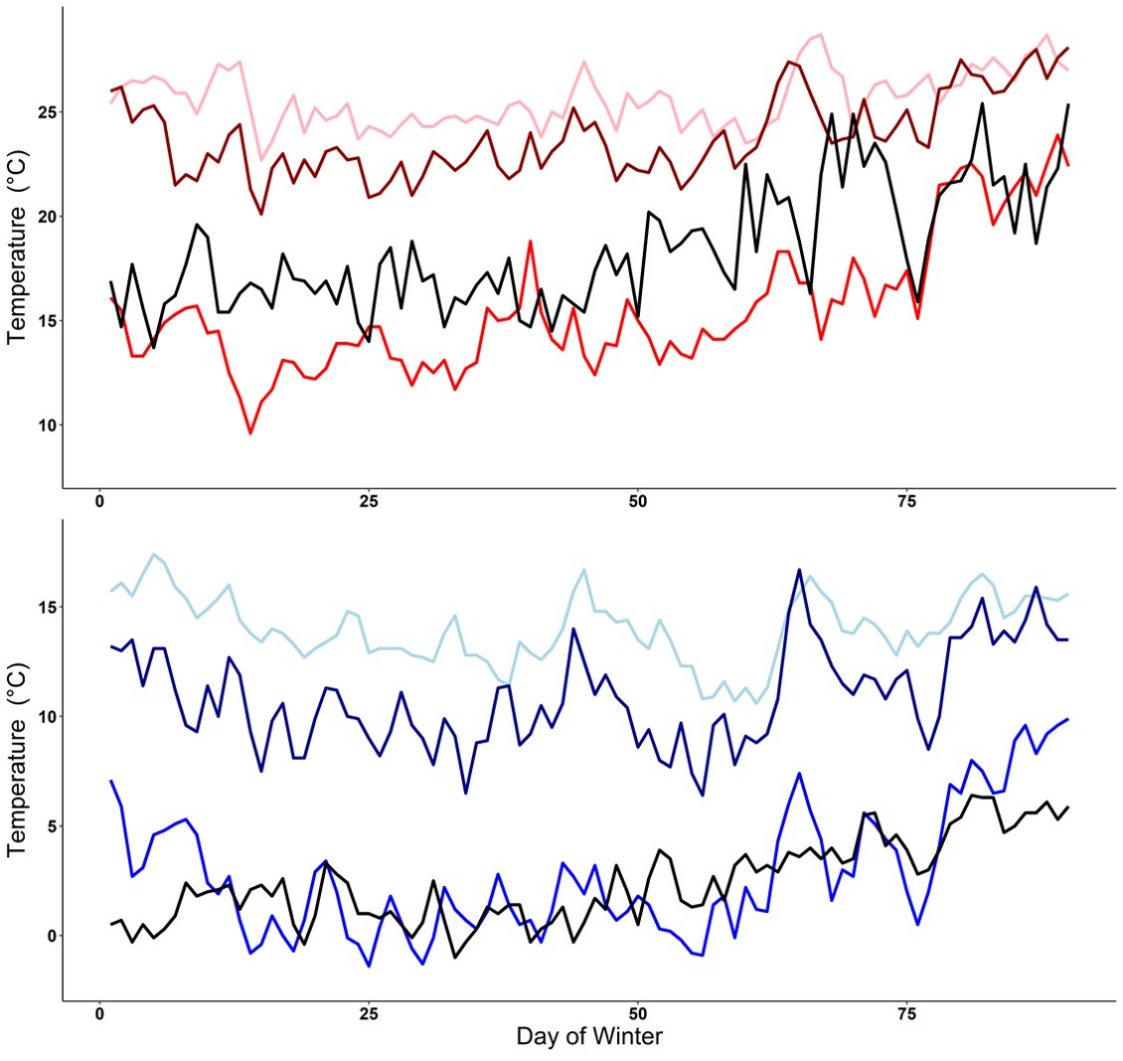
Comparison of ten-year mean air temperatures. Mean ten-year (2007–2016) daily high (top panel) and low (bottom panel) winter temperatures for Lee County, AL (study site; red line, top panel; blue line, bottom panel), Miami-Dade County, FL (invasive population; pink line, top panel; light blue line, bottom panel), Hillsborough County, FL (invasive population; dark red line, top panel; dark blue line, bottom panel), and near the southern limit of the native distribution in Santa Rosa, Argentina (black line, top and bottom panels). Includes winter period of 21 December– 20 March for the three North American sites and 21 June– 20 September for the South American location. Climate data retrieved from the National Oceanic and Atmospheric Administration (NOAA; https://www.ncdc.noaa.gov/cdo-web/datatools/selectlocation) in January 2020 from weather stations in Lee County, Alabama, USA (Station USC00010425), Hillsborough County, Florida, USA (Station USC00087205), Miami-Dade County, Florida, USA (Station USC00084095), and October 2020 for Santa Rosa, Argentina (Station AR000087623).

Daily high and low temperatures in Auburn, AL during the year of our study fluctuated in comparison to the 10-year average for this location. Most daily temperatures in the year of our study fell within the 95% confidence intervals of the 10-year average (Fig 5). However, during an extended period in January 2018 most daily temperatures were colder than average and outside ten-year confidence intervals, including 11 days with temperatures below 0°C. Moreover, snowfall occurred on 17 January 2018 with an accumulation of approximately 10 cm; accumulating snow is rare in this region of Alabama. Conversely, in February 2018 temperatures were warmer than 95% confidence intervals for an extended portion of the month. Qualitative comparison of the ten-year average daily high and low winter temperature patterns at the four sites revealed temperatures in Lee County, Alabama and Santa Rosa, Argentina were similar and routinely > 10°C colder than the two Florida locations (Fig 6).

## Discussion

Previous modeling exercises suggested wide swaths of the southern United States have suitable temperature regimes for Argentine Black and White Tegus [29]; herein we present empirical evidence that adult animals can survive under largely natural conditions in environments colder than where they currently occur. By the end of the study, all lizards that survived were healthy and appeared to be physiologically prepared for reproduction the following spring. Given the information presented here and assuming young-of-the-year lizards exhibit similar rates of winter survival as we observed in adults, we suggest there is little reason on behavioral or physiological grounds to believe tegus will be unable to survive and possibly establish populations outside of southern Florida.

Our study animals responded to environmental and physiological cues to begin and end dormancy in a manner consistent with ectotherms native to temperate climates. Tegus in Alabama remained dormant for considerably longer than the three- to four-month range reported in their native range of Brazil [32,47] or the invasive population in Miami-Dade County, FL [40]. Dormancy in the native range can last up to six months [48], but to our knowledge, our observation of a 244 d (> 8 months) dormancy period is the longest reported hibernation for this species. In a sample from southern Florida that included only males, McEachern et al. [40] reported a mean dormant period of 137 d (range = 116–160); a length of dormancy that was more than a month shorter than the 164-d average we observed here. Males in the native range exhibit a shorter period of dormancy compared with females, emerging early and competing for territories to secure copulation opportunities with resident females [41]. This pattern was repeated in Alabama, with males remaining dormant for an average of 36 fewer days and emerging 19.3 days earlier than females. Overall, we suggest variation in the length of tegu hibernation among different climatic regimes reveals a behavioral flexibility that may facilitate invasion.

During the dormancy period, we observed a mean tegu T_b_ of 14.4°C that was lower than mean values of 17–21°C [47,49] reported for dormant lizards in the native range. The physiological and ecological significance, if any, of tegu hibernation at slightly lower T_b_ is difficult to interpret because the relationship between tegu T_b_ during dormancy and survival has not been fully explored. Gold Tegus (*Tupinambis teguixin*), for example, survived overnight T_b_ as low as 7–8°C [50] and here we recorded T_b_ as low as 6.6°C in an individual that survived; it is unclear how tegus might fare if subjected to extended bouts under these thermal conditions. We did not examine survival of young-of-the-year tegus but find little reason why this segment of the population would differ in minimum thermal tolerance. Lower lethal temperatures are rarely examined [51] and these data are not available for tegus. However, lower critical minimum temperature is either not related or positively related to body size in squamates [but see 52–55] and lizards specifically [51,56]. In general, tegus appear to be physiologically capable of withstanding longer periods of dormancy at colder T_b_ than reported in Florida, but more information is needed on the lower thermal tolerance of tegus to make well-supported predictions on geographic areas at risk of invasion.

During active periods, our finding that average T_b_ was greater than ambient air temperature supports our casual observations of active thermoregulation. We observed a maximum daily T_b_ of 32–38°C during the active and late activity periods. This temperature range is consistent with observations for lizards in the native range [32,47] indicating that environmental conditions did not limit the ability of tegus to behaviorally elevate T_b_ to the sex- and size-independent preferred temperature of 36.2°C [57].

Several tegus died during our study, but our survival rate of 0.58 is similar to mean rates, and within 90% credible intervals, for two and three-plus year-old tegus in southern Florida, estimated from expert opinion [27]. Moreover, tegus in our study may have been stressed because they were not given an opportunity to adapt or acclimate to captivity or climatic conditions in Alabama that differ from those in southern Florida where they were collected. Two tegus, for instance, refused to eat offered food for the entirety of the study, one of these failed to emerge from dormancy and the other emerged in late winter but later developed a fatal respiratory infection. Both individuals were likely more prone to illness because chronic stress and poor nutrition have been shown to suppress immune response [58–60]; however, our study was not designed to investigate stress levels. Our objective was to evaluate tegu behavior and survival in a climate colder than southern Florida; notably, removal of these two likely-stressed individuals from our analyses would have resulted in a survival rate of 0.70.

The short-term emergence of two tegus in December could be evidence of thermoregulatory stress; however, this behavior may be common when hibernating in artificial refugia [47,49] and was regularly observed for some free-ranging individuals in southern Florida [8,61]. Winter conditions during this study were generally within or lower than the 95% confidence intervals for the ten-year daily average temperatures for Lee County, Alabama. December and January included extended periods that were colder than the ten-year average and it seems reasonable that a greater proportion of tegus would survive in warmer years. Climate change is predicted to increase temperature and precipitation in the region where our study occurred, and we suggest modeled effects [62] will result in increasing climatic suitability rather than decreasing the chances of survival. However, warmer temperatures do not appear to be necessary as evidenced by the high survival rate we observed in Lee County AL, and when considering populations in southern Argentina that experience similar or slightly warmer average winter temperatures.

Tegu reproduction typically occurs just after emergence from dormancy, but upon egress from refugia boxes our study animals were either at or slightly below minimum size estimates for sexual maturity [26,43], indicating they may have been too small to breed. It is also possible that we waited too long following emergence to pair males with females. We suggest reproduction would have been likely the following spring if the experiment had continued. First, cooler spring temperatures and longer winter dormancy periods would likely cause tegus to delay, rather than forego, reproduction because reptiles exhibit strong plasticity in their reproductive cycles [63–65]. Second, our study animals grew rapidly during the 2018 active period and were well above minimum size estimates for sexually maturity by late August when the study concluded. Third, post-euthanasia histological investigation of reproductive systems revealed sperm in the testes of males indicating they were capable of breeding upon emergence but were caged with females lacking eggs to ovulate. Female follicles were previtellogenic or early vitellogenic with no atretic follicles suggesting preparation for their first reproductive bout the following year. Finally, average fat pad mass accounted for 7–11 percent of total body mass; this is above a published threshold of 5% [66] deemed necessary to maintain metabolism for a twelve-month hibernation period.

All tegus euthanized at the end of the study were determined to be in good health. One female was infected with a lung parasite identified (by MAM) as the nonindigenous pentastome, *Raillietiella orientalis*, that appears to have been introduced to Florida by invasive Burmese pythons, *Python bivittatus* [67]. This female exhibited the smallest mass increase of lizards that survived the full fourteen-month study, although this individual’s mass still increased by 135%. *Raillietiella orientalis* has been observed in tegus as well as a number of native reptiles in southern Florida [67,68] but is not known from Alabama, indicating the tegu was likely infected at time of its capture in Florida. Miller et al. [68] found approximately half of native snake species in southern Florida are infected with *R*. *orientalis* and documented the spread of *R*. *orientalis* outside the range of its python host through spillover. The presence of this pentastome, and its survival in a tegu host for over a year, suggests that expansion of the invasive range of tegus may facilitate the spread of this introduced parasite into native taxa.

## Conclusions

Tegus are invasive species of great concern in the southeastern United States. In the current study we generate empirical and experimental data to complement recent modeling efforts and incidental observations, all of which point to a potential for tegus to expand well outside their currently documented range in Florida. Invasive ectotherms may not be as confined to subtropical regions as commonly thought and many factors (e.g., suitable overwintering sites, human-mediated habitat changes, reproductive plasticity) can contribute to range expansion. We suggest land managers and policy makers within the southeastern United States consider the potential for tegus to become established within their region and evaluate the merits of early-detection systems.

## Supporting information

S1 Appendix Description of radio transmitter implant surgeries and tegu necropsies. (DOCX)

S1 Fig Gross and histologic images of tegu reproductive organs. Gross and histologic images of male (Sm-7) and female (Sm-3) Argentine Black and White Tegus (*Salvator merianae*) reproductive organs. Photographs of gross structures taken on 23 August 2018 following euthanasia. Panel A shows a testicle adjacent to the adrenal gland and panel B is a histologic image of seminiferous tubule in that testicle, demonstrating normal progression of spermatogenesis containing spermatogonia, spermatocytes, spermatids and mature spermatozoa (arrowhead). Panel C shows an ovary posterior to the adrenal gland and panel d is a histologic image of previtelline follicles in that ovary. Follicles have collapsed during histologic processing. Multiple small previtelline follicles may be static preceding vitellogenesis.
(DOCX)

S1 Table Tegu capture locations in Florida. Location of the eleven traps used to capture the twelve Argentine Black and White Tegus (*Salvator merianae*) used in this study. Live traps were staged along the C-111 canal in Miami-Dade County, Florida and baited with chicken eggs.
(DOCX)
