# Supplementary material for: Argentine Black and White Tegu (Salvator merianae) can survive the winter under semi-natural conditions well beyond their current invasive range
Source: PLoS One. 2021 Mar 10;16(3):e0245877. doi: 10.1371/journal.pone.0245877 (PMC7946314; doi:10.1371/journal.pone.0245877)
Supplement: S1 Appendix — (DOCX) [file pone.0245877.s001.docx]

**S1 Appendix.** **Description of radio transmitter implant surgeries and tegu necropsies.**

Radio transponders and ibuttons were sterilized with hydrogen peroxide gas prior to surgical implantation. Tegus were manually restrained immediately prior to surgery, mass measurements were obtained, and a physical examination was performed. All Argentine Black and White Tegus (*Salvator merianae*) were apparently healthy with no abnormalities noted, save for evidence of prior tail autotomy in some individuals. Anesthesia was induced via i.v. injection (5-7 mg/kg) of propofol (PropFlo^®^ Zoetis Inc, Kalamazoo, MI 49007). Once anesthetized, tegus were intubated with a 3.0-3.5 mm Cole endotracheal tube, with anesthesia maintained with Isoflurane gas (IsoFlo^®^ Zoetis Inc, Kalamazoo, MI 49007) at 1-2% and an oxygen flow rate of 0.75 L/min using Bain nonrebreathing circuit. Supplemental manual ventilation was provided at a rate of 3-6 breaths per minute as needed. The right lateral flank was aseptically prepped using Nolvasan surgical scrub and isopropyl alcohol. An approximately 2 cm vertical incision was made between the scales approximately 1 cm caudal to the last rib. The transponder and temperature buttons were placed in the right ventral coelom. Each was secured to the body wall with a single simple interrupted suture using 3-0 polydioxaone suture (PDSII, Ethicon Inc., Bridgewater, NJ, USA). The coelomic cavity was closed using 3-0 polydioxanone suture in a simple continuous pattern in the muscle and subcutaneous layers, and the skin was opposed with an everting horizontal mattress pattern. The anti-inflammatory drug Meloxicam (Metacam solution for injection, Boehringer Ingelheim, Vetmedica, Inc St Joseph MO 64506) was injected subcutaneously at a dose of 0.3 mg/kg. Visual examinations were performed at 1, 2, and a complete physical examination was performed at 4 weeks post-surgery. All tegus returned to normal feeding within 3 d of surgery and all incisions demonstrated evidence of nearly complete healing at the time of physical examination. At the conclusion of the study, all remaining tegus were euthanized via intracoelomic injection of sodium pentobarbital + phenytoin (Euthasol, Virbac AH, Inc. Ft Worth, TX 76161) at a dose of 100 mg/kg into the ventral abdominal vein or intravenously into the ventral tail vein if the abdominal vein was not accessible.

This study was conducted in strict accordance with the recommendations and protocol approval of the Auburn University Institutional Animal Care and Use Committee (IACUC Protocol 2017-3086). All surgery was performed under propofol and isoflurane gas anesthesia, and all efforts were made to minimize suffering. Collection of Argentine Black and White Tegus was approved under a Florida Fish and Wildlife Conservation Commission permit (EXOT-17-05a). Any use of trade, firm, or product names is for descriptive purposes only and does not imply endorsement by the U.S. Government.
