# Supplementary material for: Argentine Black and White Tegu (Salvator merianae) can survive the winter under semi-natural conditions well beyond their current invasive range
Source: PLoS One. 2021 Mar 10;16(3):e0245877. doi: 10.1371/journal.pone.0245877 (PMC7946314; doi:10.1371/journal.pone.0245877)

**S1 Fig.** **Gross and histologic images of tegu reproductive organs.** Gross and histologic images of male (Sm-7) and female (Sm-3) Argentine Black and White Tegus (*Salvator merianae*) reproductive organs. Photographs of gross structures taken on 23 August 2018 following euthanasia. Panel A shows a testicle adjacent to the adrenal gland and panel B is a histologic image of seminiferous tubule in that testicle, demonstrating normal progression of spermatogenesis containing spermatogonia, spermatocytes, spermatids and mature spermatozoa (arrowhead). Panel C shows an ovary posterior to the adrenal gland and panel d is a histologic image of previtelline follicles in that ovary. Follicles have collapsed during histologic processing. Multiple small previtelline follicles may be static preceding vitellogenesis.


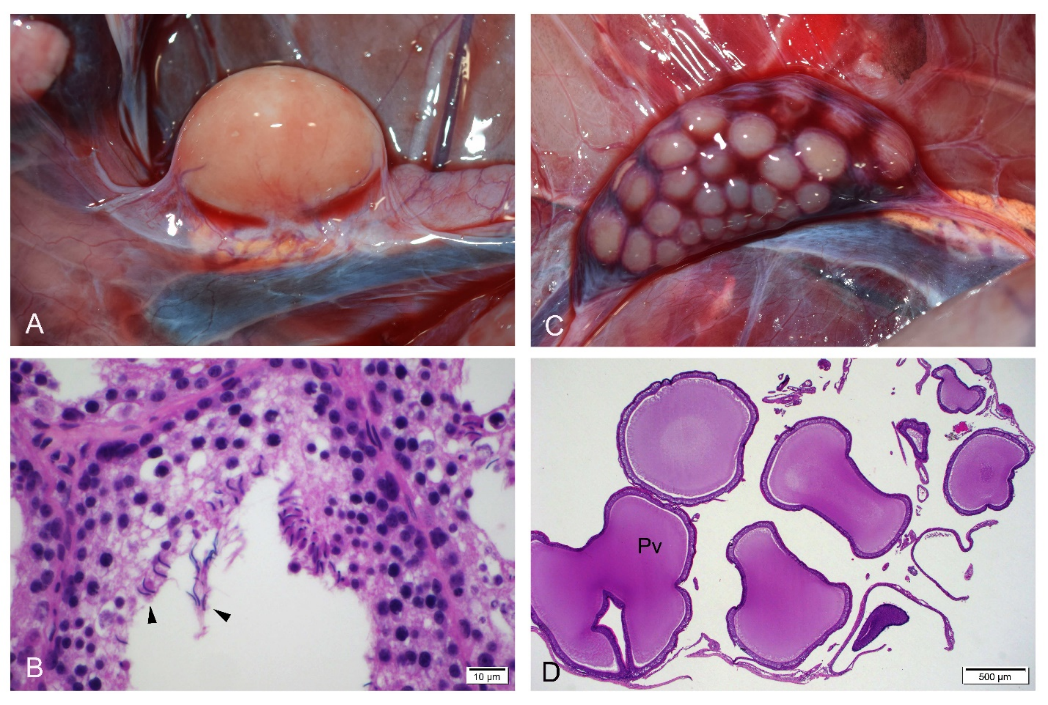

Supplement: S1 Fig — Gross and histologic images of male (Sm-7) and female (Sm-3) Argentine Black and White Tegus (Salvator merianae) reproductive organs. Photographs of gross structures taken on 23 August 2018 following euthanasia. Panel A shows a testicle adjacent to the adrenal gland and panel B is a histologic image of seminiferous tubule in that testicle, demonstrating normal progression of spermatogenesis containing spermatogonia, spermatocytes, spermatids and mature spermatozoa (arrowhead). Panel C shows an ovary posterior to the adrenal gland and panel d is a histologic image of previtelline follicles in that ovary. Follicles have collapsed during histologic processing. Multiple small previtelline follicles may be static preceding vitellogenesis. (DOCX) [file pone.0245877.s002.docx]
