# Supplementary material for: Argentine Black and White Tegu (Salvator merianae) can survive the winter under semi-natural conditions well beyond their current invasive range
Source: PLoS One. 2021 Mar 10;16(3):e0245877. doi: 10.1371/journal.pone.0245877 (PMC7946314; doi:10.1371/journal.pone.0245877)
Supplement: S1 Table — Location of the eleven traps used to capture the twelve Argentine Black and White Tegus (Salvator merianae) used in this study. Live traps were staged along the C-111 canal in Miami-Dade County, Florida and baited with chicken eggs. (DOCX) [file pone.0245877.s003.docx]

**S1 Table. Tegu capture locations in Florida.**

| Latitude | Longitude |
| --- | --- |
| 25.4226255 | -80.55918925 |
| 25.4321527 | -80.55923415 |
| 25.4020686 | -80.55797148 |
| 25.3819345 | -80.55324349 |
| 25.3899678 | -80.55794597 |
| 25.3778835 | -80.52555488 |
| 25.4131616 | -80.55917394 |
| 25.3819729 | -80.52511145 |
| 25.3653722 | -80.52988754 |
| 25.3796725 | -80.54884820 |
| 25.3817398 | -80.52565910 |

Location of the eleven traps used to capture the twelve Argentine Black and White Tegus (*Salvator merianae*) used in this study. Live traps were staged along the C-111 canal in Miami-Dade County, Florida and baited with chicken eggs.
